# Comparison of Lumbar Laminectomy Alone, Lumbar Laminectomy and Fusion, Stand-alone Anterior Lumbar Interbody Fusion, and Stand-alone Lateral Lumbar Interbody Fusion for Treatment of Lumbar Spinal Stenosis: A Review of the Literature

**DOI:** 10.7759/cureus.5691

**Published:** 2019-09-18

**Authors:** Manan Shah, Bradley Kolb, Emre Yilmaz, Dia R Halalmeh, Marc D Moisi

**Affiliations:** 1 Neurosurgery, Wayne State University, Detroit Medical Center, Detroit, USA; 2 Neurosurgery, Rush University Medical Center, Chicago, USA; 3 Surgery, Swedish Neuroscience Institute, Seattle, USA; 4 Neurosurgery, Detroit Medical Center, Detroit, USA

**Keywords:** spine, lumbar stenosis, lumbar spine fusion, lumbar laminectomy, anterior lumbar interbody fusion, lateral lumbar interbody fusion

## Abstract

Lumbar spinal stenosis is defined as narrowing of the lumbar spinal canal, which causes compression of the spinal cord and nerves. Spinal stenosis can cause leg pain and potentially back pain that can affect the quality of life. Ultimately, surgical decompression is required to alleviate the symptoms. In this review, we first utilize several important studies to compare lumbar laminectomy alone versus lumbar laminectomy and fusion. We also compare the effectiveness of more novel surgical approaches, stand-alone anterior lumbar interbody fusion (ALIF), and stand-alone lateral lumbar interbody fusion (LLIF). These techniques have their own advantages and disadvantages in which many factors must be taken into account before choosing a surgical approach. In addition, the patient’s anatomy and pathology, lifestyle, and desires should be analyzed to help determine the ideal surgical strategy

## Introduction and background

Lumbar spinal stenosis is a condition caused by narrowing of the lumbar spinal canal from hypertrophy of facets, hypertrophy of the ligamentum flavum, and/or bulging intervertebral discs, which can lead to compression of spinal nerves, cauda equina, and/or blood vessels [1]. It typically causes leg pain and potentially back pain, particularly when walking (known as intermittent neurogenic claudication), and it can severely affect one’s quality of life [2]. It is one of the most common spinal conditions in the elderly (especially older than 60) in the United States, and by 2025, about 64 million elderly will be affected by it [1,3]. Along with lumbar stenosis, concomitant lumbar degenerative spondylolisthesis can also occur, which is the slippage of one lumbar vertebra on another. Initial treatment in patients with lumbar stenosis with or without spondylolisthesis is usually conservative management including anti-inflammatory drugs, analgesics, physiotherapy, and epidural infiltration [1]. However, surgery is indicated in patients who develop refractory back and/or leg pain, or even weakness, and those who failed conservative management [1,4]. The two main approaches to surgical intervention include decompression of neural structures alone versus decompression plus fusion of adjacent vertebrae. Neural structure decompression via laminectomy has been supplemented more frequently with lumbar fusion to prevent future instability and deformity. In recent years, approximately half the patients who have had surgical intervention for lumbar stenosis have also undergone lumbar fusion [2]. There have been several studies in the past comparing the outcomes of decompressive lumbar laminectomy alone versus laminectomy with added fusion, but the results are conflicting [1]. Since the use of lumbar spine surgery has increased in the past decade, there have also been many new advances and approaches in performing lumbar fusion [2]. The five main approaches to lumbar interbody fusion include posterior lumbar interbody fusion (PLIF), transforaminal lumbar interbody fusion (TLIF), oblique lumbar interbody fusion/anterior to psoas (OLIF/ATP), anterior lumbar interbody fusion (ALIF), and lateral lumbar interbody fusion (LLIF). Each technique has its own advantages and disadvantages but there is no definitive evidence for one approach being better than the other in terms of fusion or clinical outcome [5]. In this article, we review the literature to compare the efficacy of the surgical options for lumbar stenosis, including decompressive lumbar laminectomy alone, lumbar laminectomy and fusion, stand-alone ALIF, and stand-alone LLIF.

## Review

Materials and methods 

Peer reviewed, English-only articles were searched through the PubMed database using search terms ‘lumbar stenosis, lumbar laminectomy versus fusion, stand-alone anterior lumbar interbody fusion, stand-alone lateral lumbar interbody fusion’. The inclusion criteria were as follows: original articles regarding the different surgical approaches used to address lumbar stenosis including lumbar laminectomy alone, lumbar laminectomy and fusion, stand-alone ALIF, and stand-alone LLIF. The exclusion criteria included articles that reported none of the previously mentioned correlations, those focusing on other surgical approaches used to treat lumbar stenosis, duplication among the database, and non-English language literature. The articles were then filtered to include full text review articles or clinical trial articles only.

Results

Over 5,000 articles were retrieved using these initial search terms mentioned previously. After overviewing the abstracts and titles of selected articles and filtering, duplicates were excluded from the selected pool and additional articles were removed after a re-screening of titles. The remaining articles were scrutinized for the required information. After further review, 23 peer-reviewed articles were used for this literature review to discuss lumbar laminectomy alone versus lumbar laminectomy and fusion, stand-alone ALIF, and stand-alone LLIF as follows: 

Decompressive Lumbar Laminectomy Alone vs. Lumbar Laminectomy and Fusion

Many studies have investigated whether decompression with fusion resulted in better outcomes than decompression alone for lumbar spinal stenosis. Forsth et al. conducted the Swedish Spinal Stenosis Study, in which patients were randomized to decompression alone or decompression plus fusion to treat lumbar spinal stenosis with or without degenerative spondylolisthesis [2]. The data showed that there was no significant difference between the two groups with regard to the primary outcome, using the Oswestry Disability Index (ODI) at two years and five years (ODI score ranges from 0 to 100, with higher scores indicating more severe disability) [2]. Even analyzing the subgroup of patients with and without spondylolisthesis did not show any significant difference in outcome between the groups. Visual-analogue scales (VAS) for back and leg pain were secondary outcome measures, and they also did not show any significant difference between the two groups at two years [2]. There were also no significant between-group differences with respect to required follow-up surgery within a mean period of 6.5 years, with 22% of patients in the fusion group and 21% of patients in the decompression-alone group [2]. While there were not major between-group differences in terms of efficacy or follow-up surgery, there were important differences in terms of complications and cost of care. In terms of complications, dural tears occurred at the same rate in both groups, but wound infection requiring antibiotics but not debridement was more than twice as common in the fusion group (10%) compared to the decompression-alone group (4%). The mean length of hospitalization was almost twice as long in the fusion group (7.4 days versus 4.1 days), and the fusion group also had a longer operative time, greater blood loss, and a greater cost of surgery [2]. Taken together, the results of the Swedish Spinal Stenosis Study favor simple decompression over decompression plus fusion for lumbar stenosis with or without spondylolisthesis.

Ghogawala et al. conducted the Spinal Laminectomy versus Instrumented Pedicle screw fusion (SLIP) study, a randomized controlled study that focused on a homogenous population of patients with nonmobile single-level grade 1 spondylolisthesis [6]. The patients were randomized to decompression alone or decompression plus fusion and followed for four years. The primary outcome measure looked at health-related quality of life, which was measured using the Short Form-36 (SF-36) physical component summary score (range, 0 to 100, with higher scores indicating better quality of life) [6]. It was found that at two years after surgery, patients in the fusion group had a significantly greater increase in the SF-36 physical-component summary score than did those in the decompression-alone group. This effect was sustained for three and four years after surgery [6]. However, the ODI score, a secondary measure, did not show a significant difference between the two groups at 2, 3, or 4 years post-surgery. Another important finding was that the reoperation rate over the four-year post-surgical period was 14% in the fusion group and 34% in the decompression-alone group; this difference was on the threshold for statistical significance (*P *= 0.05) [6]. The reoperations for the decompression-alone group were all for clinical instability and all the reoperations in the fusion group were for the adjacent-level disease [6]. Blood loss, length of procedure, and length of hospital stay were all significantly greater in the fusion group. The authors concluded that lumbar laminectomy plus fusion was associated with a slightly greater but clinically meaningful improvement in physical health-related quality of life compared to laminectomy alone [6].

The Swedish Spinal Stenosis study and the SLIP study have conflicting results on the efficacy of decompression alone vs. decompression and fusion, with the latter presenting evidence for the superiority of decompression plus fusion, at least for patients with symptomatic stenosis and nonmobile single-level grade 1 spondylolisthesis. The SLIP study has been criticized for its small sample size, as only 66 patients were randomized [7]. The study also showed no difference in the ODI score between the groups, which questions the conclusion that the fusion group had a better quality of life. The ODI score was also a secondary outcome measure instead of a primary one, which has also been criticized [7]. The 34% reoperation rate in the decompression-alone group is also surprisingly high, and the surgical technique has therefore been called into question by some authors [7]. The reoperation rate of the fusion group was cited at 14%, which was lower than expected. In Epstein’s 2015 and 2016 reviews of old and new literature, an adjacent level disease was seen in up to 30% of patients who underwent fusion, and reoperation rates approached 80% at five years after surgery [7]. The authors of the SLIP trial argue that ODI scores may become better over time. The change in ODI score from baseline for the two groups at four years was on the verge of statistical significance, with a *p*-value of 0.05 [6,8]. However, they state that the lack of power is a major concern for focusing on ODI scores when analyzing the two groups [8]. On the other hand, the SLIP study authors argue that the Swedish study population was heterogenous and did not identify which patients had instability or the number of levels treated, raising concern that their findings of "no benefit" could have been influenced by these shortcomings [8]. In summary, the Swedish study provides level II evidence stating that there is no difference between the decompression-alone group and the decompression plus fusion group. The SLIP study, on the other hand, provides level I evidence for the efficacy of fusion to improve outcomes and lower reoperation rates compared to the decompressive laminectomy alone for patients with spinal stenosis and nonmobile single-level grade 1 spondylolisthesis [8].

Ahmed et al. also performed a meta-analysis of randomized controlled trials and retrospective and prospective cohort studies looking at decompression alone versus decompression plus fusion for lumbar stenosis. The results showed that decompression plus fusion was found to be 2.55 times better compared to decompression alone in terms of ODI [1]. The decompression plus fusion group was found to be 2.1 times superior to the decompression alone group in terms of the VAS for back pain and 1.4 times superior for leg pain [1]. Overall, the authors concluded that decompression plus fusion is 3.5 times superior to decompression alone in terms of ODI and VAS for back pain and leg pain [1]. 

**Table 1:** Results of the comparison between lumbar laminectomy alone vs laminectomy and fusion in terms of outcome measures VAS, visual analogue scale; ODI, Oswestry disability index [1-2,6]

	Swedish Spinal Stenosis Study	Spinal Laminectomy versus Instrumented Pedicle screw fusion trial (SLIP) study	Ahmed et al. meta-analysis
ODI	No significant difference between decompression alone and decompression plus fusion	No significant difference between decompression alone and decompression plus fusion	Decompression plus fusion 2.55 times better
VAS for back pain and leg pain	No significant difference between decompression alone and decompression plus fusion	No significant difference between decompression alone and decompression plus fusion	Decompression plus fusion 2.1 times better for back pain and 1.4 times better for leg pain
Reoperation	No significant difference between decompression alone and decompression plus fusion	Greater in decompression alone group. Threshold for statistical significance (P = 0.05)	N/A
Operative Time	Significantly longer in the fusion group	Significantly longer in the fusion group	N/A
Blood Loss	Significantly greater in the fusion group	Significantly greater in the fusion group	N/A
Cost of Surgery	Significantly greater in the fusion group	Significantly greater in the fusion group	N/A
Length of Hospital Time	Significantly longer in the fusion group	Significantly longer in the fusion group	N/A

Yavin et al. performed a meta-analysis on studies comparing nonoperative management, decompression alone, and decompression plus fusion for the degenerative lumbar disease. They concluded that improvements in pain, disability, and satisfaction were greatest in patients undergoing fusion for spondylolisthesis [9]. The complications and reoperative risk limited the role of fusion in patients without spondylolisthesis [9]. Resnick et al. performed a literature review and published guidelines regarding lumbar fusion in patients without spondylolisthesis. They concluded that in the absence of instability, lumbar fusion has not been shown to improve outcomes in patients with isolated lumbar stenosis, and therefore it is not recommended [10]. In summary, there have been many studies comparing decompression alone to decompression and fusion for lumbar stenosis, but they have had varying conclusions. The patient’s anatomy, pathology, lifestyle, and desires all need to be taken into account when choosing the best option.

Stand-alone ALIF

When lumbar interbody fusion is indicated in a patient, the decision must be made on which approach to utilize safely and effectively. The anterior approach to the lumbar spine for interbody fusion is one of the predominant techniques for the surgical treatment of discogenic lumbar pain [11]. Historically, ALIF has been linked with high intraoperative complications and failure of fusion due to inadequate cages [11]. With the advent of new operative techniques and cages, stand-alone ALIF surgery has been shown to have satisfactory rates of complication and fusion. The advantages of ALIF over other approaches include direct midline view of the disc space and extensive lateral exposure of the vertebral bodies, which allows efficient disc space clearance and maximization of implant size and surface area [5]. It also allows sparing of the posterior spinal muscles and anterolateral psoas muscles, which reduces postoperative pain and disability [5]. Disadvantages include complications such as vascular and visceral injury, and retrograde ejaculation [5]. The overall risk of vascular injury is between 2.2% to 6.7%, risk of visceral injury is 5%, and the risk of retrograde ejaculation and sympathetic dysfunction is 3% [12,13]. The ALIF procedure is a good option for the L5-S1 level and a reasonable one for the L4-5 level based on the bifurcation of the great vessels [5]. It is not an option for levels above L4-5.

Rao et al. performed a prospective analysis of patients with low-grade spondylolisthesis who underwent stand-alone ALIF. They found that preoperative spondylolisthesis was reduced to 6.4% postoperatively, and disc height was increased to 175% of preoperative values, with both differences reaching statistical significance [14]. The VAS pain score improved from 7.6 to 2.2 and ODI improved from 56.9% to 17.8%, with both differences reaching statistical significance [14]. The radiological fusion rate was 91%. The overall clinical success rate was 93% [14]. Lammli et al. reviewed a series of patients with degenerative lumbar disc disease who underwent a level 1 or 2 stand-alone ALIF. At two-year follow-up, the ODI and VAS scores significantly improved over presurgical levels in the ALIF patients [15]. No patient experienced intraoperative or major complications. Out of the 118 patients, three had reoperations not related to the adjacent level or fusion site, three had reoperations related to adjacent level disease, and three had pseudoarthrosis at the fusion level [15]. Amaral et al. performed a retrospective single-center study looking at patients with lumbar stenosis and grade 1 spondylolisthesis who underwent L5-S1 stand-alone ALIF. VAS for back pain decreased from 7.4 preoperatively to 4.2 at three months, and VAS for lower limbs decreased from 5.1 preoperatively to 2.8 at three months, with both differences reaching statistical significance [11]. ODI decreased from 44 preoperatively to 31 at three months, which was also statistically significant [11]. Out of 87 patients, two had venous damage intraoperatively, and two had an accidental opening of the peritoneum. There was one patient with postoperative retroperitoneal hematoma and one with incisional hernia, but no patients had retrograde ejaculation [11]. There have not been many studies regarding stand-alone ALIF surgery for lumbar stenosis but since the advances in interbody cages, it has shown very promising results.

Stand-alone LLIF

The lateral approach to the lumbar spine is a novel technique described by Ozgur et al. which consists of accessing the disc space via a lateral retroperitoneal and transpsoas surgical corridor [5,16]. This approach can be utilized for approaching the lateral spine from the T12-L1 level to the L4-L5 level [5]. It is not suitable for the L5-S1 level because of obstruction by the iliac crests [5,13]. Benefits of LLIF include a large discectomy, bilateral annular release, insertion of large grafts, correction of deformity, and indirect decompression of spinal nerves [17]. Compared to the anterior approach, this approach is less invasive and avoids retraction of the great vessels and sympathetic chain [13]. The lateral approach, unlike the anterior approach, also typically preserves the ligamentous structures including the anterior longitudinal ligament [14,17-19]. Obesity can facilitate the lateral approach to the target disc by pulling the peritoneal contents anteriorly; therefore, resulting in an easier approach through the retroperitoneal corridor [13]. Conversely, obesity can be a barrier to success with the anterior approach used in ALIF. Relative contraindications to LLIF include abnormal or challenging vascular or plexus anatomy as well as previous retroperitoneal surgery [5,13]. With LLIF, there are potential risks to the lumbar plexus, psoas muscle, kidneys and bowel, and sufficient care must be taken during the approach to avoid these key structures [5,13,17].

Ahmadian et al. performed a multicenter chart review study for patients who underwent stand-alone minimally invasive LLIF, and 59 patients were ultimately included with pathologies such as degenerative disc disease, spondylolisthesis and scoliosis [20]. The fusion rate was 93% at 12 months and only two patients needed a reoperation [20]. The VAS improved from 69.1 to 37.8 and ODI improved from 51.8 to 31.8, with both differences reaching statistical significance [20]. Of note, 70% of patients had grade 0 subsidence, while 30% had grade I and II subsidence [20]. Marchi et al. conducted a prospective observational study of 52 patients who underwent stand-alone LLIF for single-level grade I/II spondylolisthesis. Data showed that the mean VAS back scores decreased from 78 to 31 and mean VAS leg scores decreased from 54 to 31, with both differences reaching statistical significance [18]. The mean ODI scores also significantly improved from 66% to 30% [18]. Fusion was seen in 86.6% of levels treated where incomplete bone ingrowth was observed in the remainder; however, pseudoarthrosis was not seen. Postoperatively, there were 10 patients (19.2%) who presented with psoas weakness and five patients (9.6%) who had anterior thigh numbness, but both conditions resolved within six weeks [18]. There were a total of seven levels (13.5% of cases) where revision surgery was necessary. There were five revision cases due to high-grade subsidence causing instability or restenosis and two revisions because decompression was not achieved [18]. Agarwal et al. performed a retrospective analysis on 55 patients over the age of 70 undergoing a stand-alone LLIF. The ODI score significantly decreased from 46.2 to 31.1 [21]. Five patients needed to undergo revision surgery for graft subsidence [21]. The study concluded that stand-alone LLIF can be safely and effectively performed in the elderly. Watkins et al. found a non-union rate of 19% per level and 27% per patient [17]. Nemani et al. performed a retrospective analysis on 117 patients who underwent stand-alone LLIF and found that at 16-month follow-up, 10.3% required revision surgery for posterior decompression, mostly for restenosis [22]. The authors concluded that stand-alone LLIF represents an acceptable procedure in patients with lumbar spinal stenosis. 

Laws et al. compared the biomechanical differences between stand-alone ALIF and stand-alone LLIF. Compared to the intact state, stand-alone LLIF significantly reduced the range of motion in flexion, extension, and lateral bending [13,23]. On the other hand, the authors found that stand-alone ALIF did not stabilize motion segments relative to intact state [13,23]. Overall, the stand-alone LLIF procedure can be utilized effectively in certain patient populations. 

**Table 2:** Results of comparison between stand-alone ALIF and stand-alone LLIF ALIF, anterior lumbar interbody fusion; LLIF, lateral lumbar interbody fusion; ODI, Oswestry disability index; VAS, visual analogue scale [11-12,15,17-18,20-21]

	Stand-alone ALIF	Stand-alone LLIF
Advantages	Direct midline view of the disc space and extensive lateral exposure of the vertebral bodies, which allows efficient disc space clearance and maximization of implant size and surface area Sparing of the posterior spinal muscles	Large discectomy, bilateral annular release, insertion of large grafts, correction of deformity, and indirect decompression of spinal nerves. Less invasive, and avoids retraction of the great vessels and sympathetic chain. Sparing of the posterior spinal muscles and anterior longitudinal ligament (ALL). Obesity can help with approach by pulling the peritoneal contents anteriorly
Disadvantages	Risk of vascular and visceral injury, and retrograde ejaculation. Does not preserve anterior longitudinal ligament. Obesity can be a barrier to successful approach.	Can cause injury to lumbar plexus. Can cause psoas muscle weakness. Risk of vascular injury as well as injury to kidney and bowl/colon.
ODI - Preoperative to Postoperative	56.9% to 17.8%, statistically significant (Rao et al.) Improved significantly at two years (Lammli et al.). 44% to 31% at 3 months, statistically significant (Amaral et al.)	51.8% to 31.8%, statistical significant (Ahmadian et al.). 66% to 30 %, statistically significant (Marchi et al.). 46.2% to 31.1%, statistically significant (Agarwal et al.)
VAS for back pain and leg pain Preoperative to Postoperative	7.6 to 2.2, statistically significant (Rao et al.) Improved significantly at 2 years (Lammli et al.). 7.4 to 4.2 for leg pain at 3 months, 5.1 to 2.8 for back pain at 3 months, both statistically significant (Amaral et al.)	69.1 to 37.8, statistically significant (Ahmadian et al.). 78 to 31 for back pain, 54 to 31 for leg pain, both statistically significant (Marchi et al.)
Fusion rate	91% (Rao et al.)	93% (Ahmadian et al.), 86.6% (Marchi et al.)
Reoperation rate	5% (Lammli et al.)	3.3% (Ahmadian et al.), 13.5% (Marchi et al.), 9.1 % (Agarwal et al.), 10.3% (Watkins et al.)

## Conclusions

Lumbar spinal stenosis is a condition that can greatly impact a patient’s quality of life. Many studies have been done comparing decompressive lumbar laminectomy alone versus decompression and fusion for lumbar stenosis. In general, lumbar laminectomy alone has been shown to be better for patients with lumbar stenosis in the absence of instability. Studies have shown that lumbar decompression and fusion has been better in patients with spondylolisthesis. In patients who require a fusion, stand-alone ALIF has shown promising results due to the recent advancements in cages, especially at the L4-5 and L5-S1 levels. Stand-alone LLIF has also shown to be an acceptable procedure for lumbar spinal stenosis. Overall, each technique for the treatment of lumbar stenosis has its own advantages and disadvantages. The individual patient, pathological process, and his or her anatomy must be reviewed to decide which surgical approach is the best.
